# Discovery of the marine Eocene in the northern South China Sea

**DOI:** 10.1093/nsr/nwz084

**Published:** 2019-06-27

**Authors:** Zhimin Jian, Haiyan Jin, Michael A Kaminski, Fabricio Ferreira, Baohua Li, Pai-Sen Yu

**Affiliations:** 1 State Key Laboratory of Marine Geology, Tongji University, China; 2 Geosciences Department, King Fahd University of Petroleum and Minerals, Saudi Arabia; 3 Institute for Geosciences, Universidade Federal Fluminense, Brazil; 4 Nanjing Institute of Geology and Paleontology, Chinese Academy of Sciences, China; 5 Applied Research Laboratories (NARLabs), Taiwan Ocean Research Institute (TORI), China

The nature of the Eocene stratigraphy of the South China Sea (SCS) has been a mystery for decades. In the southern SCS, the Eocene system with marine deposits is often considered as representing part of the ‘Proto-South China Sea’ [1]. In the northern SCS, however, the Eocene is dominated by non-marine sequences of South China origin [2], probably also related to the Proto-SCS [3]. Then the question is what kind of SCS northern margin was in the Eocene: part of the South China continent or part of Proto-SCS or even an eastern extension of the shrinking Tethys Ocean? Furthermore, the significance of the Eocene issue is enhanced by the recent debates on the opening age of the modern SCS, whether in the Eocene or in the early Oligocene [4]. The existence of the marine Eocene in the northern SCS was implied by reworked Eocene nannofossils found in offshore oil exploration wells over 20 years ago [5] by Eocene foraminifers and nannofossils found in cutting samples from deep-water oil wells [6] and by Eocene marine deposits reported recently from Taiwan [7]. Until now, however, solid direct evidence of marine Eocene depositions in the northern SCS has been absent. Here, we report new discoveries of deep- and shallow-water marine Eocene sediments based on the results of the International Ocean Discovery Program (IODP) drilling in the northern margin of the SCS.

## STRATIGRAPHY

**Figure 1:**
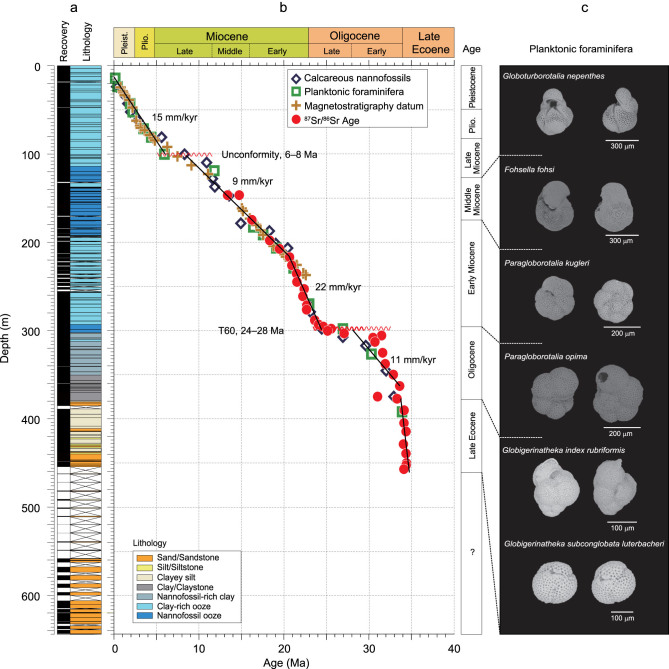
Lithostratigraphic, biostratigraphic, magnetostratigraphic [8] and strontium isotope stratigraphic correlations at Site U1501. (a) Lithology; (b) depth-age plot of Site U1501 based on the shipboard biostratigraphic data of calcareous nannofossils and planktonic foraminifera, magnetostratigraphic data [8] and the Sr isotopic ages from this study; numbers beside the lines indicate the average sedimentation rates. Red zigzag lines represent the unconformities, while T60 indicates the seismic reflector; (c) SEM photographs of index planktonic foraminifera including typical late Eocene species.

A continuous terrestrial to deep-sea sedimentary record of the Eocene and younger ages was obtained for the first time at Site U1501 (18°53′N, 115°46′E, water depth 2846 m) in the northern SCS margin during the IODP Expedition 368 [8]. Planktonic foraminifera and calcareous nannofossils are abundant in the upper 300 m of the sediment section and common to rare between 300 and 460 m, but barren below 460 m, indicating a transition from terrestrial to shallow-marine to deep-marine facies. In the core catcher sample 54X-CC, we found typical late Eocene planktonic foraminiferal assemblage comprising *Globigerinatheka index rubriformis* and *Globigerinatheka subconglobata luterbacheri* [9] (Fig. 1c) and a late Eocene calcareous nannofossil assemblage comprising *Coccolithus formosus*, which has a last appearance datum older than 32.9 Ma, *Discoaster nodifer*, *Discoaster tanii* and *Reticulofenestra umbilicus* as well as other Eocene species [8]. Therefore, the marine sediments below ∼392 m (i.e. below the mid-point between samples 53X-CC and 54X-CC) represent the late Eocene deposition. The results of ^87^Sr/^86^Sr measurement based on the calibration method of [10] also show a trend similar to that of biostratigraphy and magnetostratigraphy at this site (Fig. 1b; detailed in the ‘Methods’ section and Supplementary Data), further supporting the assignment of the marine sediments below 380 m in the core to the late Eocene, older than 34 Ma. Unlike the biostratigraphic datums, however, Sr-isotope stratigraphy cannot provide precise geological ages due to the influence of terrestrial input and a certain degree of fossil recrystallization.

**Figure 2:**
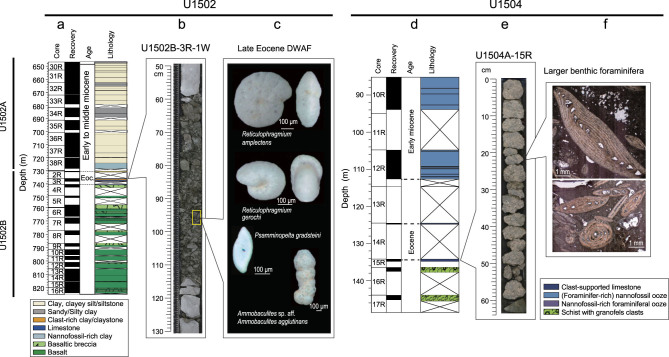
Comparison of marine Eocene fossils at Sites U1502 and U1504 (modified from [8]). (a) Lithology of Site U1502; (b) core image of U1502B-3R-1 W; (c) SEM photos of selected DWAF species (at the depth of 734.69 m); (d) lithology of Site U1504; (e) core image of U1504A-15R; (f) thin-section photographs of larger benthic foraminifera (at the core depth of 134.43 m).

At the seaward Site U1502 (18°28′N, 116°14′E, water depth 3764 m), the basement rock is altered basalt, above which are deep-sea deposits with abundant nannofossils and planktonic foraminifera of late Oligocene and younger ages. In the topmost part of the highly altered basalt, there is an interval of alternating metasediments (dolomite marble, dolomitic limestone and clast-rich clay), from which a sandy sample (734.69 m; 94–99 cm in Section 1 of Core 3R, Hole B) yielded abundant and diverse deep-water agglutinated benthic foraminifera (DWAF) without any calcareous species [8]. The DWAF species found at this site include *Reticulophragmium amplectens*, *Reticulophragmium gerochi* and *Psamminopelta gradsteini* (Fig. 2c). This ‘Flysch-type’ assemblage with similar DWAF was previously reported in Eocene strata from the North Atlantic and western Tethys, as well as from the neighboring Sulawesi Sea [11]. The comparison of the DWAF found in this study with the previously reported cosmopolitan species [12] indicates an age of late Eocene, constraining the age of the topmost altered basalt at Site U1502.

Marine Eocene fossils were also found at Site U1504 (18°51′N, 116°15′E, water depth 2817 m), which is located on the outer margin high of the northern SCS like Site U1501 mentioned above. Below 112.7 m, at Site U1504, is a reef limestone that is 10–20 m thick in which planktonic foraminifera and calcareous

nannofossils are barren, but these microfossils are abundant in the overlying carbonate ooze of the early Miocene and younger ages. However, thin sections of the carbonate rocks from 134.43 m (21–23 cm in Section 1 of Core 15R, Hole A) contain abundant larger benthic foraminifera such as *Nummulites* and *Assilina* (Fig. 2f), similar to those found from the Eocene strata of the Philippine Archipelago [13], suggesting an Eocene age for the limestone [8]. Therefore, late Eocene or older marine sediments were recovered at all the three IODP sites U1501, U1502 and U1504 (the micropaleontological data have been documented at http://publications.iodp.org/proceedings/367_368/367368title.html, which would provide closer insight into the distribution/abundance patterns of the key microfossils), although the detailed stratigraphic correlation between them needs further work.

## PALEO-WATER DEPTH

Paleo-water-depth reconstruction is helpful for understanding the relative amplitude of subsidence and marine

transgressions associated with the formation of the SCS basin. At Site U1501, the clastic facies below 460 m contains no marine microfossils indicating a terrigenous origin. Up-core from 460 to 380 m, in the late Eocene sediments, foraminifera are common to rare and dominated by shallow-marine benthic species including *Planularia* sp., *Gaudryina* sp. and *Quinqueloculina* sp., similar to those found in the marine Eocene syn-rift sediments of Taiwan [7]. Benthic foraminifera even constitute about 98% of the total foraminiferal abundance for this interval, indicating a very shallow-marine environment on the continental shelf. The ostracoda assemblage is characterized by upper bathyal and continental-shelf taxa (e.g. *Cytherelloidea*, *Cytheropteron*, *Xestoleberis* and *Cytherella*), although deep-sea genus *Krithe* remains present [8]. The co-existence of deep- and shallow-water benthic foraminifera and ostracoda shells suggests that the sediment likely accumulated by a re-sedimentation process in a location not far away from the coast. At Site U1504, the Eocene coral-reef deposit is directly covered by deep-water early Miocene to Recent deep-sea sediments [8], indicating a hiatus >10 Ma in duration. Therefore, we speculate that the outer margin high of the northern SCS was a shallow-marine continental shelf setting during the late Eocene.

**Figure 3:**
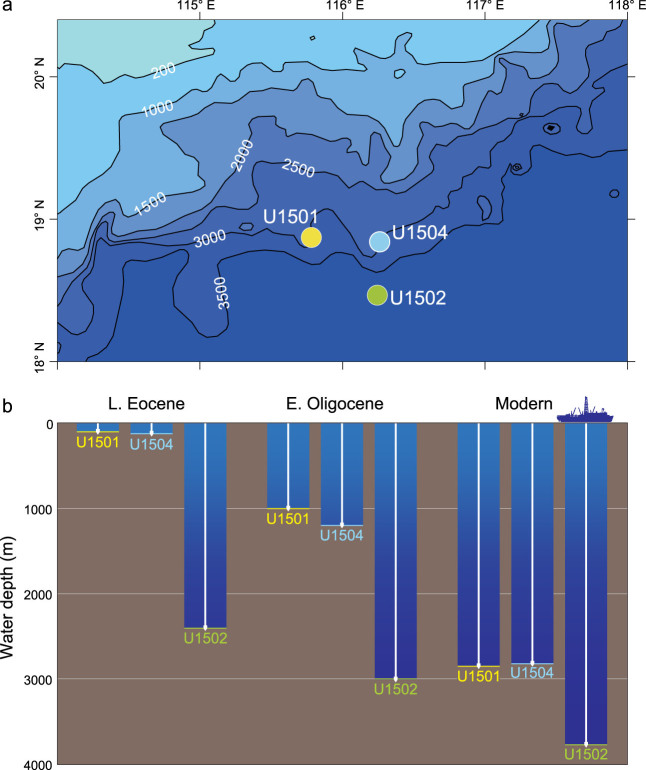
Bathymetric changes in the north SCS. (a) Modern water depth of the drill sites; (b) schematic diagram showing water-depth changes at these sites since the late Eocene.

At Site U1502, however, the late Eocene DWAF assemblage dominated by species living at water depths between 1000 and 3500–4000 m [12] indicates a paleo-water depth below the carbonate compensation depth (CCD). At present, the SCS CCD lies at about 3500 m, which is 1100 m shallower than that of the open Pacific Ocean. A previous study based on carbonate-accumulation records estimates deepening of the open Pacific CCD from 3500–4000 m during the middle and late Eocene to 4600 m in the Oligocene [14]. By comparison, we infer that the paleo-CCD of the northern SCS during the late Eocene was about 2400–2900 m. This explains the occurrence of the DWAF assemblage was likely due to the relative shallow paleo-CCD. Therefore, Site U1502 was bathed in very deep water during the late Eocene, whereas Sites U1501 and U1504 were mainly influenced by shelf environments. As Site U1502 now lies only about 69.6 and 42.7 km farther offshore from Sites U1501 and U1504, respectively, the late Eocene shelf in this area must be considerably narrow compared to the wide shelf of the modern northern SCS (up to ∼500 km) (Fig. 3).

The early Oligocene sediments at Site U1501 contain relatively abundant benthic foraminifera with many shallow-water marker species, indicating an outer shelf to upper slope environment at a water depth shallower than 1000 m, when compared with the modern analogues of the SCS. This inference is also supported by the finding of many pteropods in the early Oligocene at ODP Site 1148 of the northern SCS [15], because these microfossils can be only found from above the modern aragonite compensation depth at 1000∼1200 m.

In the late Oligocene, the paleo-water depth of the SCS remarkably deepened, more drastically from above T60, the most prominent seismic reflector in the Paleogene SCS [16]. According to our high-resolution Sr-isotope stratigraphy, the end age of T60 is 24.0 Ma, but its initial age varies from ∼30 Ma (based on

Sr-isotope stratigraphy) to ∼28 Ma (based on biostratigraphy), indicating a sedimentary hiatus of at least 4-Ma duration (Fig. 1b). Apparently, T60 represents a major tectonic movement close to the Oligocene/Miocene boundary. Immediately above T60 at Site U1501 saw a sudden jump in the planktonic foraminiferal abundance to >95%, indicating a water-depth increase to 2500 m or more [15]. This subsidence of >2000 m is also witnessed at Site U1504, where the Eocene coral-reef deposit is directly covered by the early Miocene deep-sea sediment [8]. Therefore, all the outer margins of the northern SCS became part of the SCS deep-sea basin since the early Miocene.

## TECTONIC IMPLICATIONS

The new Eocene data from the IODP Expedition 368, as described above, provide direct evidence of marine deposition in the northern SCS margin, yet they are insufficient to answer the question about the nature of marine influence in relation to the wider tectonic framework in the Eocene SCS. It remains unclear, for example, whether the discovered Eocene sediment belongs to the earliest deposition of the SCS basin or to the remnant Proto-SCS or both. If the SCS opening started from the east and then propagated westward since the Eocene with its oldest oceanic crust already subducted beneath the Manila trench, the newly discovered Eocene deposits can be considered as evidence to support an Eocene initiation of the SCS basin. But, if the Proto-SCS persisted into the Eocene, then these deposits would represent its northern margin marine record in an Eocene Proto-SCS that also sustained in its southern part the accumulation of such strata as the deep-marine turbidite Rajang Group [17] and Crocker Fan sandstones [18] in northern Borneo. Much further work is needed to clarify these issues.

Nevertheless, the discovery of the marine Eocene in the northern SCS margin has profound tectonic and paleogeographic implications. About 20 years ago, deep-water Oligocene deposits recovered at ODP Site 1148 confirmed the prevalent speculation that the SCS was formed 32–33 Ma ago [19]; now, the DWAF assemblage found at U1502 provided robust evidence for the existence of deep-water environments even earlier in the Eocene. Deep-water conditions at the rift/drift transition or at the beginning of SCS sea-floor spreading is in sharp contrast to the development model of the non-volcanic passive margin in the Atlantic [20], but appears to characterize the newly supposed plate-edge rifting model [21].

Microfossil data also pose a biogeography issue, namely a possible connection of the Eocene (Proto-) SCS with the shrinking Tethys Ocean. The late Eocene DWAF at U1502 consists of cosmopolitan species that display strong affinities to coeval assemblages described from the North Atlantic, western Tethys [12] and Pacific including the Sulawesi Sea [11]. Meanwhile, *Nummulites* and other larger benthic foraminiferal genera found at Site U1504 indicate a warm, shallow-marine environment in the late Eocene or even in slightly younger age intervals [8]. Similar assemblages have been widely found in the Eocene strata of Maldives [22], the Philippine Sea [13] and the Pacific Ocean offshore of Japan [23], suggesting a close biogeographic connection along the margins of the Tethys Ocean in the Eocene [24]. Taken together, these records bear some transitional biogeographic features between the Tethys and Pacific and, hence, strongly imply that the late Eocene SCS or Proto-SCS basin was located at the junction between the two great oceans.

## METHODS

Strontium analysis of foraminiferal shells was performed on 35 samples from the interval of 140–460 m in the core (Supplementary Table 1). Approximately 20–40 shells of mixed planktonic and benthic foraminifera were picked from the size fraction >154 μm. Foraminiferal samples were pretreated following the procedure for the Mg/Ca ratio measurements and further checking was also performed under a microscope to eliminate potential contamination. The samples were dissolved in 1 ml 3 M HNO_3_ solution and then loaded into a 2-ml column filled with 50–100 μm Sr Spec resin. The samples were analysed using a MC-ICP-MS (NEPTUNE plus) at the State Key Laboratory of Marine Geology, Tongji University. ^86^Sr/^88^Sr = 0.1194 was adopted to calibrate mass bias during the Sr-isotope measurements and the NIST NBS 987 standard was repeatedly measured with the samples to monitor the quality of the measurements, yielding an average ^87^Sr/^86^Sr of 0.710242 ± 15 (2 SD) (*N* = 14). Our laboratory blanks were <500 pg. The high-resolution calibration of marine ^87^Sr/^86^Sr through time [10] was adopted in this study.

## Supplementary Material

nwz084_Supplemental_File
