# Supplementary material for: Discovery of the marine Eocene in the northern South China Sea
Source: Natl Sci Rev. 2019 Jun 27;6(5):881–5. doi: 10.1093/nsr/nwz084 (PMC8291548; doi:10.1093/nsr/nwz084)
Supplement: nwz084_Supplemental_File [file nwz084_supplemental_file.pdf]

SUPPLEMENTARY DATA

Table S1 Results of the strontium analysis of foraminiferal shells at Site U1501

Site	Hole	Core	Type	Section	Top offset (cm)	Bottom offset (cm)	CSF-A (m)	$^{86}Sr/^{87}Sr$	2SE	Age (Ma)
U1501	C	16	H	3	103	105	146.33	0.708811	0.000017	14.04
U1501	C	21	F	3	42	44	174.34	0.708716	0.000017	16.21
U1501	C	26	F	3	32	34	197.73	0.708557	0.000019	18.31
U1501	C	28	F	3	42	44	207.23	0.708466	0.000013	19.45
U1501	C	30	F	3	42	44	216.63	0.708398	0.000012	20.65
U1501	C	32	F	3	32	34	225.93	0.708383	0.000020	20.91
U1501	C	34	F	3	32	34	235.23	0.708352	0.000019	21.47
U1501	C	36	F	3	32	34	244.73	0.708349	0.000012	21.51
U1501	C	38	F	2	52	54	252.83	0.708297	0.000013	22.37
U1501	C	40	X	3	102	104	261.03	0.708306	0.000016	22.21
U1501	C	41	X	5	128	130	271.30	0.708281	0.000012	22.68
U1501	C	42	X	2	100	102	276.21	0.708282	0.000017	22.65
U1501	C	43	X	4	40	42	288.21	0.708234	0.000012	23.61
U1501	C	44	X	1	16	18	293.07	0.708213	0.000011	24.06
U1501	C	44	X	2	100	102	295.42	0.708181	0.000012	24.62
U1501	C	44	X	4	40	42	297.83	0.708136	0.000012	25.56
U1501	C	44	X	5	128	130	300.23	0.708153	0.000018	25.13
U1501	C	45	X	1	68	70	303.19	0.708083	0.000018	27.07
U1501	C	45	X	3	6	8	305.58	0.707945	0.000011	31.48
U1501	C	45	X	4	97	99	307.99	0.707974	0.000018	30.47
U1501	C	46	X	1	68	70	312.79	0.707967	0.000019	30.70
U1501	C	47	X	3	40	42	325.12	0.707942	0.000014	31.61
U1501	C	48	X	5	40	42	337.74	0.707931	0.000012	31.91
U1501	C	49	X	7	40	42	349.93	0.707892	0.000021	32.86
U1501	C	51	X	2	130	132	362.82	0.707852	0.000014	33.61
U1501	C	52	X	4	71	73	374.82	0.707959	0.000170	31.01
U1501	C	52	X	6	10	12	377.23	0.707868	0.000016	33.30
U1501	C	54	X	2	8	10	390.39	0.707824	0.000011	34.14
U1501	C	55	X	5	38	40	404.79	0.707825	0.000009	34.12
U1501	C	56	X	5	38	40	414.39	0.707813	0.000020	34.36
U1501	C	58	X	2	8	10	428.80	0.707828	0.000006	34.05
U1501	C	59	X	2	8	10	439.39	0.707814	0.000012	34.35
U1501	C	61	X	2	40	42	450.21	0.707811	0.000021	34.40
U1501	C	61	X	3	130	132	452.61	0.707813	0.000017	34.36
U1501	C	62	X	2	68	70	457.09	0.707825	0.000020	34.10
